# Clinical Significance of Pregnancies with Macrosomia (Birth Weight ≥ 4,000 g) Compared with Deliveries with Neonatal Birth Weight of 3,500-3,999 g

**DOI:** 10.31662/jmaj.2024-0117

**Published:** 2024-12-20

**Authors:** Shunji Suzuki

**Affiliations:** 1 Department of Obstetrics and Gynecology, Japanese Red Cross Katsushika Maternity Hospital, Tokyo, Japan

**Keywords:** macrosomia, shoulder dystocia, neonatal asphyxia, vaginal delivery, elective cesarean delivery

## Abstract

**Introduction:**

We examined the clinical characteristics and perinatal outcomes of pregnancy with macrosomia (birth weight ≥ 4,000 g) compared with delivery with neonatal birth weight of 3,500-3,999 g.

**Methods:**

This study analyzed data obtained from singleton pregnant women who delivered at ≥22 weeks of gestation from January 2002 to December 2010.

**Results:**

During the study period, there were 12,497 singleton deliveries, of which 136 (1.1%) had macrosomia (average: 4,181 g, range: 4,000-4,726 g; macrosomia group) and 1,139 (9.1%) had neonatal birth weight of 3,500-3,999 g (average: 3,670 g; control group). Compared with the control group, the macrosomia group had advanced maternal age and births after 41 weeks of gestation. In addition, elective cesarean delivery was more common in the macrosomia group (*P* < 0.01). Furthermore, the rate of shoulder dystocia was higher in this group in cases of vaginal delivery (*P* < 0.01). A high rate of neonatal asphyxia was also observed in the macrosomia group (*P* < 0.01), although there were no significant differences in the rate of low umbilical artery pH or the incidence of neonatal hypoglycemia between the groups. Multivariate analysis revealed that the significant complications in the macrosomia group compared with the control group were shoulder dystocia (*P* = 0.01) and neonatal asphyxia (*P* = 0.03).

**Conclusions:**

The results of this study indicate that particular attention should be paid to the possibility of shoulder dystocia during delivery of macrosomia.

## Introduction

The term “macrosomia” has been derived from the Greek words “macro,” which means big, and “somia,” which refers to the body ^(1)^. Although establishing a universally accepted definition for macrosomia is potentially challenging ^(2)^, the Japan Society of Obstetrics and Gynecology ^(3)^ defines it as neonate with a birth weight of 4,000 g or more without malformations or other gross abnormalities, regardless of the gestational weeks of birth. Macrosomia is reportedly associated with maternal and neonatal complications, such as shoulder dystocia, severe perineal laceration, and neonatal hypoglycemia ^(1), (2), (3), (4), (5), (6)^. In particular, shoulder dystocia is an obstetric complication in which normal traction on the fetal head does not lead to delivery of the shoulders ^(7)^. It can result in permanent neurologic sequalae and perinatal death. Shoulder dystocia reportedly occurs in approximately 1% of vaginal deliveries with neonatal birth weight less than 4,000 g and in 5%-10% of vaginal deliveries with neonatal birth weight of 4,000-4,500 g ^(6)^. However, most cases of shoulder dystocia seemed to have occurred without warning; thus, delivery staff should always be prepared to recognize and treat this complication ^(7)^. Therefore, it is clinically useful to know the criteria for fetal weight that would more accurately predict the occurrence of shoulder dystocia.

Based on these backgrounds, we herein examined the clinical characteristics and perinatal outcomes of pregnancy with macrosomia compared with delivery with neonatal birth weight of 3,500-3,999 g. In addition, we examined the criteria of fetal weight requiring serious delivery management in considering with the occurrence of shoulder dystocia. In other words, considering obstetric complications, such as shoulder dystocia, we determined whether the criterion of 4,000 g or more for macrosomia is appropriate for assessing obstetric risk in the Japanese population.

## Materials and Methods

This retrospective case-control study was approved by the Ethics Committee of the Japanese Red Cross Katsushika Maternity Hospital.

The study analyzed data obtained from all singleton pregnant women who delivered at the aforementioned hospital at ≥22 weeks of gestation from January 2002 to December 2010. The 8-year study period was chosen as it was the longest among the databases maintained by the same system with the same obstetric medical director. During the study period, there were 12,497 singleton deliveries at ≥22 weeks of gestation, of which 136 (1.1%) had macrosomia (birth weight ≥ 4,000 g; average: 4,181 g, range: 4,000-4,726 g; macrosomia group) and 1,139 (9.1%) had neonatal birth weight of 3,500-3,999 g (average: 3,670 g; control group). As previous studies have usually compared the perinatal outcomes of pregnancies with appropriate fetal weight and those with macrosomia, the risk of macrosomia was evaluated ^(1), (2), (3), (4), (5), (6), (7)^. However, the validity of appropriate criteria for macrosomia could not be assessed. To evaluate the risk of macrosomia, we used deliveries with neonatal birth weight of 3,500-3,999 g as the control group.

In our institute, we do not perform prophylactic induction of labor when fetal macrosomia is suspected; however, we perform an elective cesarean section with an informed consent for the possibility of shoulder dystocia ^(8), (9),(10), (11)^. During the study period, our institute, where most of the obstetricians have 3-10 years of experience and were sent from our affiliated university hospitals, did not experience any substantial changes in their environment, such as their experience level and the policies for managing fetal macrosomia according to the guidelines for obstetrical practice in Japan ^(3)^.

We excluded deliveries referred from other obstetric institutes after the onset of labor pains. This was due to the possibility of a different management for delivery with macrosomia.

Furthermore, we examined maternal age, parity, maternal height and weight before pregnancy (and the body mass index), gestational weight gain, gestational age at delivery, history of glucose intolerance, and neonatal sex as maternal clinical characteristics and delivery modes, shoulder dystocia, severe perineal lacerations, postpartum hemorrhage requiring transfusion, neonatal asphyxia, low umbilical artery pH (<7.0), and neonatal hypoglycemia as perinatal outcomes. Gestational age was calculated based on ultrasonographic findings at 9-11 weeks of gestation. Pregnancies complicated by glucose intolerance included those complicated by overt diabetes and those diagnosed with gestational diabetes mellitus (GDM). GDM was diagnosed when at least one of the following symptoms was present: fasting blood glucose level ≥92 mg/dL, blood glucose level ≥180 mg/dL at 1 h, or blood glucose level ≥153 mg/dL at 2 h in the 75-g oral glucose tolerance test during pregnancy. Shoulder dystocia was diagnosed as the failure to deliver the fetal shoulders solely using gentle downward traction following expulsion of the head ^(1)^. Severe perineal lacerations included third- or fourth-degree perineal lacerations. Neonatal asphyxia was diagnosed when the neonatal Apgar score at 1 min was less than 7 points. In this study, neonatal hypoglycemia was defined as serum blood glucose level of less than 45 mg/dL.

Data were expressed as number (percentage, %). The SAS software version 8.02 (SAS Institute, Cary, NC, USA) was used for the statistical analyses. Furthermore, chi-squared test and Fisher’s exact test were employed, with *P* < 0.05 considered to indicate statistical significance. The variables used in the multivariate model were those that had shown significant association with the occurrence of perinatal complications on univariate analysis. The odds ratios and 95% confidence intervals (CIs) were also calculated.

## Results

The clinical characteristics of the macrosomia and control groups are presented in Table 1. The incidence of macrosomia was associated with advanced maternal age and births after 41 weeks of gestation.

**Table 1.** Clinical Characteristics of Pregnancies with Macrosomia Compared with Deliveries with Neonatal Birth Weight of 3,500-3,999 g.

	Control	Macrosomia	*P*-value
Total number	1139	136	
Maternal age < 20 years	10 (0.9)	2 (1.5)	0.50
Maternal age ≥ 35 years	303 (26.6)	48 (35.3)	0.03
Nulliparity	498 (43.7)	50 (36.5)	0.12
Maternal height ≥ 1.6 m	490 (43.0)	63 (46.3)	0.46
Maternal body mass index at prepregnancy ≥ 25	387 (34.0)	55 (40.4)	0.13
Gestational weight gain ≥ 15 kg	68 (6.0)	6 (4.4)	0.46
Gestational age at delivery ≥ 41 weeks	342 (30.0)	58 (42.6)	<0.01
Glucose intolerance	26 (2.3)	6 (4.4)	0.13
Neonatal sex: male	712 (62.5)	87 (64.0)	0.74

Data are expressed as number (%)

Furthermore, the perinatal outcomes in the groups are shown in Table 2. Elective cesarean delivery was more common in the macrosomia group (*P* < 0.01) than in the control group. In addition, the rate of shoulder dystocia was higher in the former group when limited to the cases of vaginal delivery (*P* < 0.01). There was also a high rate of neonatal asphyxia in the macrosomia group (*P* < 0.01), although no significant differences were observed in the rate of low umbilical artery pH or the incidence of neonatal hypoglycemia between the groups. Multivariate analysis revealed that the significant complication in pregnancy with macrosomia compared with that with neonatal birth weight of 3,500-3,999 g were shoulder dystocia (*P* = 0.01) and neonatal asphyxia (*P* = 0.03), as shown in Table 3.

**Table 2.** Perinatal Outcomes of Pregnancies with Macrosomia Compared with Deliveries with Neonatal Birth Weight of 3,500-3,999 g.

	Control	Macrosomia	*P*-value	OR	95% CI
Total number	1139	136			
Elective cesarean delivery					
Yes	88 (7.7)	21 (15.4)	< 0.01	2.18	1.3-3.6
No	1051 (92.3)	115 (84.6)	Reference	1	
Emergent cesarean delivery					
Yes	90 (7.9)	16 (11.8)	0.12	1.55	0.9-2.3
No	1049 (92.1)	120 (88.2)	Reference	1	
Vaginal delivery					
Yes	961 (84.4)	99 (72.8)	< 0.01	0.50	0.3-0.8
No	178 (15.6)	37 (27.2)	Reference	1	
Normal delivery	844 (/961, 87.8)	85 (/99, 85.9)	Reference	1	
Vacuum extraction/forceps delivery	100 (/961, 10.4)	8 (/99, 8.1)	0.47	0.76	0.4-1.6
Shoulder dystocia	3 (/961, 0.3)	4 (/99, 4.0)	< 0.01	13.24	3.3-52.4
Severe perineal lacerations	14 (/961, 1.5)	2 (/99, 2.0)	0.65	1.42	0.4-5.6
Postpartum hemorrhage requiring transfusion					
	6 (0.5)	2 (1.5)	0.19	2.82	0.7-12.4
	1133 (99.5)	134 (98.5)	Reference	1	
Perinatal death					
Yes	1 (0.1)	0 (0)	0.73	0	0-32.3
No	1138 (99.9)	136 (100)	Reference	1	
Neonatal asphyxia					
Yes	15 (1.3)	10 (7.4)	< 0.01	5.95	2.7-13.3
No	1124 (98.7)	126 (92.6)	Reference	1	
Umbilical artery pH					
≥7.0	1133 (99.5)	134 (98.5)	Reference	1	
<7.0	6 (0.5)	2 (1.5)	0.19	2.82	0.7-12.4
Neonatal hypoglycemia					
Yes	98 (8.6)	19 (14.0)	0.06	1.73	1.0-2.9
No	1041 (91.4)	117 (86.0)	Reference	1	

OR: odds ratio, 95% CI: 95% confidence interval. Data are expressed as number (%)

**Table 3.** Results of the Multivariate Analysis of the Perinatal Outcomes of Pregnancies with Macrosomia.

	*P*-value	Adjusted OR	95% CI
Elective cesarean delivery	0.13	1.56	0.9-2.8
Vaginal delivery	0.28	0.80	0.6-1.2
Shoulder dystocia in vaginal delivery	0.01	7.11	1.8-29.1
Neonatal asphyxia	0.03	3.03	1.1-8.2

OR: odds ratio, 95% CI: 95% confidence interval.

A summary of the seven cases of shoulder dystocia is shown in Table 4. Of these cases, seven (71%) were parous women. Only one case (14%) had an umbilical artery pH <7, whereas four cases (57%) had neonatal asphyxia. None of these cases resulted in cerebral palsy or neurological sequelae, such as brachial plexus injury ^(9)^.

**Table 4.** Summary of 7 Cases of Shoulder Dystocia.

Case	Maternal age (y)	Parity	Gestational age at delivery (w + d)	Delivery mode	Neonatal sex	Birth weight (g)	Apgar score at 1 min	Umbilical artery pH
1	32	1	40 + 4	Normal delivery	Male	3,878	6	7.251
2	33	0	42 + 0	Forceps delivery	Male	3,898	7	7.240
3	29	2	39 + 2	Normal delivery	Female	3,902	1	7.271
4	36	1	39 + 5	Normal delivery	Male	4,024	9	7.238
5	35	2	41 + 6	Normal delivery	Male	4,118	4	7.278
6	33	2	41 + 3	Normal delivery	Male	4,290	2	7.239
7	32	0	36 + 6	Normal delivery	Male	4,436	1	6.949

## Discussion

We examined the clinical characteristics and perinatal outcomes of pregnancy with macrosomia compared with pregnancy with neonatal birth weight of 3,500-3,999 g. We believe that the present study is significant because the medical management of pregnancies in which the estimated fetal body weight of approximately 3,500 g or more must actually take into account the possibility of macrosomia ^(3)^. Macrosomia has been reported to be associated with maternal obesity, multiparity, advanced maternal age, preexisting diabetes, and developed gestational diabetes ^(5), (12), (13), (14)^. Furthermore, fetal male sex has been reported to be associated with adverse perinatal outcomes related to the occurrence of macrosomia ^(15)^. These were the risk factors for macrosomia examined among all pregnancies, whereas in this study among pregnancies with birth weight of 3,500 g or more, only maternal advanced age and advanced gestational week at delivery were the risk factors for macrosomia with significant differences.

Among all pregnancies, those with macrosomia has been reported to be associated with significant obstetric morbidities, such as prolonged labor, increased risk of instrumental vaginal delivery, shoulder dystocia, severe perineal laceration, emergency cesarean delivery, postpartum hemorrhage, neonatal asphyxia, and admission to the neonatal intensive care unit ^(5), (12), (13), (14), (15)^. However, among the deliveries with neonatal birth weight of 3,500 g or more in this study, macrosomia was associated with shoulder dystocia leading to neonatal asphyxia. The findings of the umbilical artery blood gas suggest that neonatal asphyxia in cases of shoulder dystocia was caused by fetal stress just before delivery, as shown in Table 4. Although none of these cases were ultimately problematic, shoulder dystocia is a well-known complication that can cause neonatal death or serious sequelae ^(1), (14), (16)^. During the study period, no shoulder dystocia occurred in deliveries with neonatal birth weight of 3,500 g or lower in our institute (unpublished data). Shoulder dystocia can occur even in fetuses with no overgrowth ^(2), (7)^. However, the results of the present study indicate that particular attention should be paid during delivery of fetuses weighing approximately 3,900 g or more.

In our institute, prophylactic induction of labor when fetal macrosomia has not yet been performed. Although there have been some reports that labor induction for macrosomia decreases the rate of cesarean section, particularly among multiparous women ^(17), (18)^, we currently have no intention of changing our current policy. In a previous study ^(10)^, labor induction in cases of ultrasonic estimation of fetal weight between 4,000 and 4,500 g did not decrease the rate of cesarean delivery or shoulder dystocia. The current evidence in the USA also does not support a policy for early labor induction before 39 weeks of gestation or delivery for suspected macrosomia ^(2), (19)^. In this study, shoulder dystocia occurred in 71% of parous women. Therefore, we believe that informed consent for elective cesarean delivery to prevent shoulder dystocia is considered acceptable in cases of macrosomia, as previously reported ^(20), (21)^.

This study has several limitations that need to be acknowledged. First, this study has a very small sample size. Thus, we could not perform sufficient statistical examination as there were only seven cases of shoulder dystocia. As discussed in the Methods section, we preferred to examine the same delivery-controlled subjects and did not adequately consider the sample size, although the need for a large-scale study was understood. Second, we did not perform ultrasonographic examination to detect macrosomia. The diagnosis of macrosomia through ultrasound fetal measurements is challenging ^(22)^, and without the studies ^(22)^, it may be difficult to further investigate ways to improve the adverse outcomes of macrosomia. Third, the blood glucose levels of the macrosomia were periodically measured after birth; however, the measurement was not necessary in the control group if the neonates were not heavy-for-date and were feeding well. Therefore, a large prospective study based on the results of ultrasound fetal measurements concerning the delivery mode for macrosomia may be necessary.

### Conclusion

Among pregnancies with neonatal birth weight of 3,500 g or more, maternal advanced age and advanced gestational week at delivery were the risk factors for macrosomia with perinatal complications. Furthermore, macrosomia was associated with shoulder dystocia leading to neonatal asphyxia. The current results also indicated that particular attention regarding the possibility of shoulder dystocia should be paid during delivery of fetuses weighing approximately 3,900 g or more.

In the obstetric management of high-weight fetuses, evaluation of fetal weight will be required according to individual obstetric complications, rather than delimiting the estimated fetal weight of 3,500 or 4,000 g or more.

## Article Information

### Conflicts of Interest

None

### Author Contributions

Shunji Suzuki: project development, data management, data analysis, and manuscript writing and editing.

### Approval by Institutional Review Board (IRB)

The study protocol was approved by the Ethics Committee of the Japanese Red Cross Katsushika Maternity Hospital (K2017-20).

### Informed Consent

Patients’ informed consent for the publication of this report was obtained.
